# Meldonium Supplementation in Professional Athletes: Career Destroyer or Lifesaver?

**DOI:** 10.7759/cureus.63634

**Published:** 2024-07-01

**Authors:** Amalia Pușcaș, Mădălina-Georgiana Buț, Camil-Eugen Vari, Bianca-Eugenia Ősz, Ruxandra Ștefănescu, Cristina Filip, George Jîtcă, Tudor-Ionuț Istrate, Amelia Tero-Vescan

**Affiliations:** 1 Biochemistry, Faculty of Pharmacy, George Emil Palade University of Medicine, Pharmacy, Science and Technology, Târgu Mureș, ROU; 2 Pharmacology and Clinical Pharmacy, Faculty of Pharmacy, George Emil Palade University of Medicine, Pharmacy, Science and Technology, Târgu Mureș, ROU; 3 Pharmacognosy and Phytotherapy, Faculty of Pharmacy, George Emil Palade University of Medicine, Pharmacy, Science and Technology, Târgu Mureș, ROU

**Keywords:** doping, performance athletes, carnitine inhibitor, metabolic modulators, meldonium

## Abstract

Meldonium is a substance with known anti-anginal effects demonstrated by numerous studies and human clinical trials; however, it does not possess marketing authorization within the European Union, only in ex-Soviet republics. Since 2016, meldonium has been included by the World Anti-doping Agency (WADA) on the S4 list of metabolic modulators. In performance athletes, meldonium is now considered a doping agent due to its capacity to decrease lactate production during and after exercise, its capability to enhance the storage and utilization of glycogen, and its protective action against oxidative stress. Together, these attributes can significantly improve aerobic endurance, cardiac function, and capacity as well as shorten recovery times (allowing higher intensity training), thereby enhancing performance.

The purpose of this review is to highlight the most important mechanisms underlying the protective effect of meldonium against mitochondrial dysfunction (MD), which is responsible for oxidative stress, inflammation, and the cardiac changes known as "athletic heart syndrome." Meldonium acts as an inhibitor of γ-butyrobetaine hydroxylase (BBOX), preventing the de novo synthesis of carnitine and its absorption at the intestinal level via the organic cation/carnitine transporter 2 (OCTN2) and directing the oxidation of fatty acids to the peroxisomes. The decrease in mitochondrial β-oxidation of fatty acids leads to a reduction in lipid peroxidation products that cause oxidative stress and prevent the formation of acyl/acetyl-carnitines involved in numerous pathological disorders.

Given the recent findings of the potentially detrimental effects of prolonged high-intensity exercise on cardiovascular health and coronary atherosclerosis, there may be legitimate arguments for the justification of the use of substances like meldonium as protective supplements for athletes.

## Introduction and background

The use of illicit substances to enhance athletic performance is a subject that continuously challenges the authorities. The World Anti-doping Agency (WADA) includes several classes of substances on its list, depending on the type of physical effort (aerobic or anaerobic) and the benefits they provide (pain/inflammation management or athletic performance enhancement), such as analgesics and cannabinoids, steroidal anti-inflammatories, central stimulants, anabolic steroids, androgen receptor modulators, erythropoiesis stimulators, or masking agents [1].

Metabolic modulators can provide substantial benefits for athletic performance due to the judicious use of energy resources, with the advantage of possessing a much better safety profile compared to other compounds used for performance enhancement. For example, anabolic steroids produce hyperestrogenism (with gynecomastia, oligospermia, etc.) and need the concomitant use of an aromatase inhibitor to prevent the conversion of testosterone to estradiol to reduce these effects [2,3]. The risk of benign prostatic hyperplasia also increases due to the elevated levels of dihydrotestosterone (DHT), so a 5-alpha reductase inhibitor is added [4]. All these pharmacological interventions, intended to combat side effects, complicate the doping scheme. Erythropoiesis stimulators increase the risk of cardiovascular side effects as a result of stimulating erythrocyte synthesis [5], while central stimulants can cause insomnia, tremors, tachyarrhythmias, hypertension, stroke, and headache, with different pharmaco-toxicological profile depending on the compound used [6].

In the end, the question arises whether metabolic modulators truly provide such significant benefits to high-performance athletes compared to the previously mentioned classes of substances, whose beneficial effects are well documented. To answer this question, the biochemical mechanisms underlying the potential effects must be well understood.

Meldonium (mildronate), chemically known as 3-(2,2,2-trimethylhydraziniumyl)propionate, has shown promising anti-anginal and neuroprotective effects in various animal models of Alzheimer's or Parkinson’s disease or hypoxia-induced lung injury [7,8]. This substance was included together with trimetazidine on the metabolic modulators subsection list by WADA in 2016 [1,9]. Before 2016 it was on WADA’s list of monitored substances to detect abusive cliches in sport. Based on the structural similarity of meldonium with a precursor in the synthesis of L-carnitine, it has been established that it modifies the energy metabolism of fatty acids in the mitochondria. Mitochondria are a semi-autonomous cellular organelle that possesses a circular double-helical mitochondrial DNA (mtDNA), which encodes important enzymes involved in energy metabolism. They have a reparative and adaptive capacity under stress conditions (physical exertion and nutritional deficiencies) through processes of fusion and fission [10].

Physical effort can have both beneficial and negative dose-dependent effects on inflammatory processes and oxidative stress. While moderate physical effort is beneficial in reducing the risk of chronic and inflammatory diseases by modulating the activity of protein kinases such as the mammalian target of rapamycin (mTOR) or AMP-activated protein kinase (AMPK) and the serum levels of inflammatory markers such as leptin, adiponectin, ghrelin, interleukin-6 (IL6), interleukin-1β (IL1β), and tumor necrosis factor α (TNFα), strenuous physical exertion has the opposite effect, with direct repercussions on mitochondrial metabolism [11,12]. In the case of performance athletes, intense muscular exertion can sometimes cause irreversible degenerative changes in the metabolism and functionality of mitochondria, a process known as mitochondrial dysfunction (MD) [13]. MD, in turn, causes changes in energy metabolism, ATP production, the occurrence of toxic levels of reactive oxygen and nitrogen species (ROS/RNS), and the exacerbation of inflammation [14].

Considering that no negative effects of meldonium administration in athletes have been demonstrated, the present narrative review aims to elucidate the most important mechanisms through which meldonium may have a beneficial effect on MD that occurs in those who practice sports consistently, outside of competitions.

## Review

Modification of L-carnitine metabolism induced by meldonium

**Figure 1:**
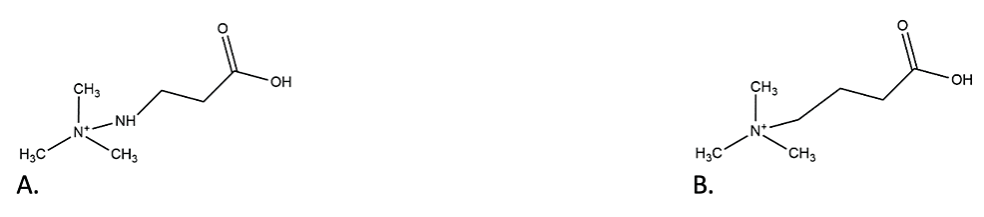
Chemical structure of meldonium (A) and GBB (B) GBB: γ-butyrobetaine. Source: This image is the original work of the authors, and the image was created by ChemDraw.

Meldonium is a structural analog of γ-butyrobetaine (GBB) (Figure 1), a precursor compound in L-carnitine synthesis. L-carnitine, the intramitochondrial transporter of long-chain fatty acids for β-oxidation, is synthesized through a series of four reactions, the last of which is catalyzed by γ-butyrobetaine hydroxylase (BBOX), an enzyme competitively inhibited by meldonium [15]. This mechanism of action is demonstrated through studies on male lean Zucker rats. Administration of meldonium (1.6 g/kg bw/day for two days and 0.8 g/kg bw/day for up to 10 days) led to the depletion of muscle L-carnitine deposits, a reduction in fatty acid β-oxidation, and an increase in carbohydrate utilization for energy purposes. Additionally, this study showed the potential of meldonium to modify gene expression in muscle tissue by reducing the expression of L-carnitine transporter proteins via 189 mRNAs regulating fuel selection [16].

Another mechanism of action of meldonium is its competition with L-carnitine and GBB for binding to the organic cation/carnitine transporter 2 (OCTN2). OCTN2 facilitates the significant accumulation of organic cations such as L-carnitine and GBB within tissues. Additionally, at the intestinal and renal levels, OCTN2 plays a role in the absorption of organic cations in the ileum and their reabsorption in the kidneys [17,18].

Meldonium does not influence, or only weakly inhibits, the activity of enzymes involved in the transport of long-chain fatty acids into the mitochondria. Thus, meldonium does not have a direct inhibitory effect on carnitine palmitoyltransferase-1 (CPT1). Moreover, it increases the mRNA and protein expression levels of this enzyme. It is a weak inhibitor of carnitine acetyltransferase (CrAT) in vitro and lacks any effect in vivo. Additionally, it exhibits weak competitive inhibition of carnitine/acyl-carnitine translocase (CACT), an enzyme involved in the carnitine/acyl-carnitine antiport [18].

By decreasing L-carnitine-induced mitochondrial β-oxidation of fatty acids, meldonium redirects fatty acids β-oxidation toward peroxisomes through increased gene expression of peroxisome proliferator-activated receptor γ coactivator 1α (PGC-1α) and peroxisome proliferator-activated receptor alpha (PPAR-α) [19].

In the peroxisomes, long-chain fatty acids are transformed into shorter-chain compounds, which are much easier to metabolize in mitochondria. This prevents the formation of long-chain acyl-carnitines, the accumulation of which in mitochondria is implicated in ischemic heart disease, proarrhythmic muscle contractions, and increased contractility of the myocardium [20].

**Figure 2:**
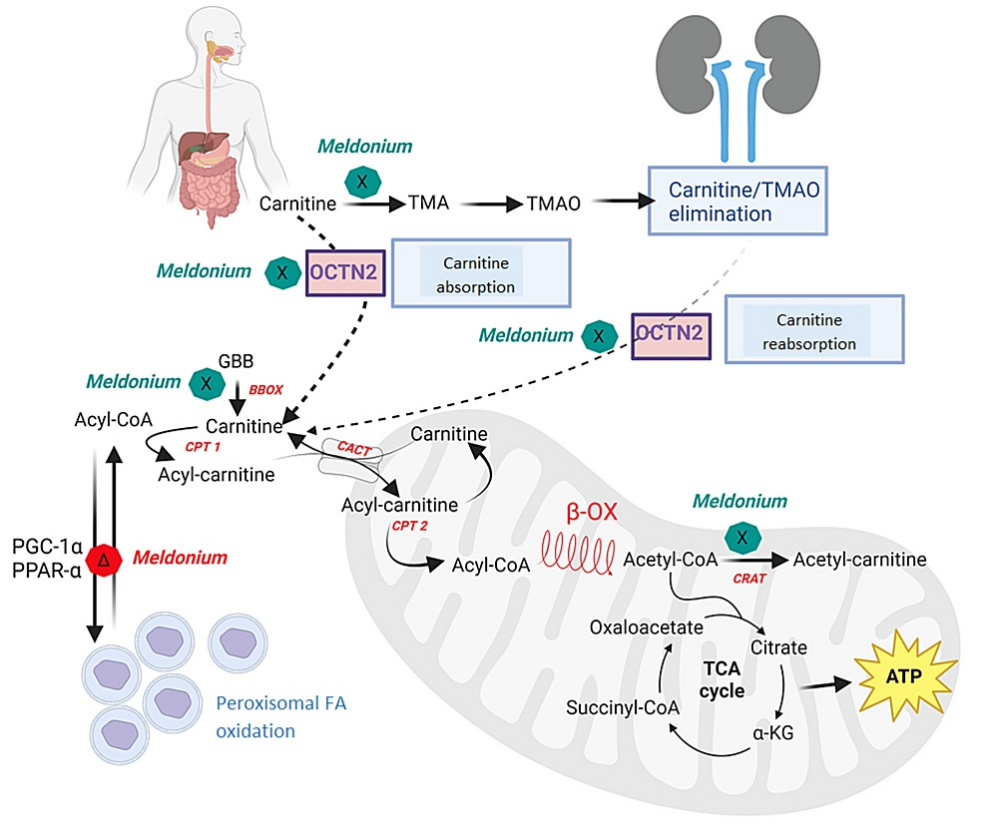
Meldonium mechanism of action In the cytoplasm, the activated fatty acid as acyl-CoA binds to L-carnitine, forming acyl-carnitine by carnitine palmitoyltransferase-1 (CPT1). Acyl-carnitine is transferred into the mitochondria under the action of carnitine/acyl-carnitine translocase (CACT). Inside the mitochondria, acyl-carnitine is converted back to acyl-CoA and L-carnitine by the action of carnitine palmitoyltransferase-2 (CPT2) and enters the process of β-oxidation (β-OX) to form acetyl-CoA. Acetyl-CoA is further metabolized in the Krebs cycle or transformed into acetyl-carnitine by the enzyme carnitine acetyltransferase (CrAT). Within the mitochondria, meldonium inhibits the CrAT enzyme, thus regulating the ratio of acetyl-CoA/acetyl-carnitine. By inhibiting the enzyme γ-butyrobetaine hydroxylase (BBOX), meldonium inhibits the synthesis of L-carnitine at the final step, the transformation of γ-butyrobetaine (GBB) into L-carnitine, thereby reducing its concentration in the cell. Fatty acids are redirected to peroxisomes where they are transported and oxidized independently of L-carnitine. Meldonium induces increased gene expression of peroxisome proliferator-activated receptor γ coactivator 1α (PGC-1α) and peroxisome proliferator-activated receptor alpha (PPAR-α). At the intestinal level, meldonium inhibits organic cation/carnitine transporter type 2 (OCTN2) and decreases the absorption of dietary L-carnitine into the body, which is then eliminated through urine. Additionally, meldonium inhibits the formation of trimethylamine (TMA) from GBB by intestinal microbiota and promotes the elimination of trimethylamine-N-oxide (TMAO) through urine. At the renal level, meldonium reduces the reabsorption of L-carnitine. Green marks signify inhibition and red marks signify activation of a pathway. Source: This image is the original work of the authors, and the image was created by BioRender.com.

Moreover, at the intestinal level, meldonium reduces the transformation of L-carnitine into trimethylamine (TMA) under the action of intestinal flora. TMA is a precursor compound of trimethylamine-N-oxide (TMAO), which has negative effects on cardiovascular health. TMAO acts as an activator of inflammatory pathways and promotes foam cell formation. It is associated with increased mortality in patients with chronic kidney disease [21]. The mechanism of action of meldonium is presented schematically in Figure 2.

Meldonium as a protector in oxidative stress-induced MD

Intense and recurrent physical exertion can have negative effects on the cardiac muscle, ranging from left ventricular hypertrophy to increased ventricular dysrhythmias and diastolic ventricular dysfunction, a phenomenon known as "athletic heart syndrome" [22]. Although there is no consensus in the scientific literature regarding the dose, intensity, and type of physical exercise that can have negative effects on cardiac metabolism, it is unanimously accepted that under these conditions, myocardial adaptation occurs through an increase in the number of mitochondria and intensification of fatty acid β-oxidation for energy purposes. Mitochondrial biogenesis is mediated by PGC-1α and activated during effort by an AMP/ATP ratio > 1 [23].

The increase in the number of mitochondria leads to overproduction of ROS, oxidative damage of mtDNA, MD, and may trigger cell death [24]. mtDNA lacks histones and presents a less efficient repair system compared to nuclear DNA. Compounds formed through lipid peroxidation, together with the depletion of other protective factors in oxidative stress such as glutathione (GSH), can cause damage to mitochondrial membranes.

A study of forced swimming (swimming for one hour/day for seven consecutive days) conducted in mice showed an increase in mtDNA concentration and a decrease in GSH, leading not only to mitochondrial biogenesis but also to an increase in free radical concentration, which led to cellular apoptotic processes in the heart. Administering meldonium to these animals reduced MD by altering energy metabolism from using fatty acid β-oxidation for energy purposes to using glucose, capable of producing ATP under hypoxic conditions associated with intense physical effort [25].

**Figure 3:**
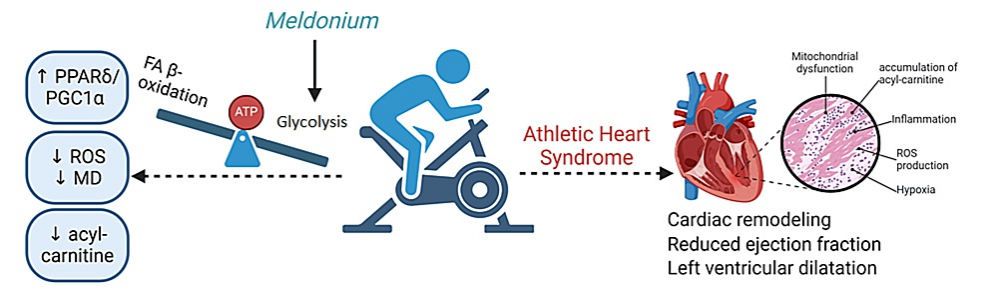
Cardioprotective effect of meldonium in hypoxic conditions induced by effort In athletes, prolonged efforts can cause athletic heart syndrome by promoting inflammation, ROS (reactive oxygen species) production, hypoxia, MD (mitochondrial dysfunction), and accumulation of acyl-carnitine. Meldonium inhibits carnitine metabolism and decreases FA β-oxidation (fatty acids beta-oxidation) in the mitochondria. At the same time, meldonium activates glucose metabolism by activating 6PF1K (6-phosphofructo-1-kinase) and pyruvate dehydrogenase and shifts the production of ATP in case of aerobic effort from lipids to carbohydrates. This mechanism of action for meldonium presents a favorable overall effect on the athletes’ heart. PPARδ: Peroxisome proliferator-activated receptor delta; PPAR-α: Peroxisome proliferator-activated receptor alpha. Source: This image is the original work of the authors, and the image was created by BioRender.com.

Another likely mechanism for meldonium to reduce oxidative stress during hypoxia-induced lung injury is the promotion of translocation of nuclear factor erythroid 2-related factor 2 (Nrf2) from the cytoplasm to the nucleus and the activation of 6-phosphofructo-1-kinase (6PF1K), the enzyme that catalyzes the rate-limiting step in glycolysis [14,26,27] (Figure 3). An experiment conducted on Swiss mice with exposed type II alveolar epithelial cells to hypobaric hypoxic conditions showed that meldonium activates 6PF1K and modulates gene expression of important enzymes in pyruvate metabolism such as the M2 isoform of pyruvate kinase, isozyme A of lactate dehydrogenase, and pyruvate dehydrogenase kinase 1. Under these conditions, meldonium administration leads to a reduction in oxidative stress, as demonstrated by decreased levels of malondialdehyde (MDA) and increased superoxide dismutase activity (SOD), thus reducing oxidative stress-induced MD caused by alterations in mitochondrial fission and fusion balance [24]. A study in an experimental Sprague-Dawley rat hypoxia model conducted in 2023 showed that the protective effect of meldonium manifests not only in the lungs but also in other organs such as the liver, brain, heart, and kidney [28].

Meldonium, the modulator of inflammation-induced MD

Hypoxia induced at high altitudes or during intense physical exertion causes a series of modifications such as polycythemia, increased levels of proinflammatory cytokines (IL1β, IL6, and TNFα) in lung tissue, and changes in parameters of oxidative stress (elevated lipid peroxidation product and protein carbonyl content as well as decreased GSH and total antioxidant capacity), all of which lead to MD [29].

In a murine model of lipopolysaccharide (LPS)-induced inflammation, meldonium reduced the extent of mtDNA damage and decreased the concentration of diene conjugates in the hippocampus in a Nrf2-independent manner, possibly due to a profound modification of energy metabolism and reduction of oxidative stress [30]. The anti-inflammatory effect of meldonium was studied in a rat model of LPS-induced sepsis. Administration of meldonium at a dose of 300 mg/kg bw/day for four weeks before inducing sepsis in animals showed an increased activity of tissue copper-zinc SOD, manganese SOD, catalase (CAT), and glutathione peroxidase (GSH-Px) and reduced levels of lipid peroxidation, TNFα, phospho-nuclear factor kappa B p65 form (p-NF-κB p65), and the Bax/Bcl-2 apoptotic marker ratio, suggesting the anti-inflammatory and anti-apoptotic effects of meldonium [31,32]. Studies on the administration of meldonium to carnivorous fish show a decrease in the gene expression of proinflammatory molecules (IL1β and TNFα) and apoptotic molecules (Caspase 3 and 9) in the liver [24,33].

The use of meldonium in pulmonary hypertension-induced right ventricular failure and inflammation-induced left ventricular systolic dysfunction, like those seen in COVID-19, shows that meldonium alone or in combination with anti-inflammatory drugs improves heart function in cases of excessive inflammation and cytokine storm, according to a study conducted on animals [34]. Treatment with meldonium in a rat model of traumatic brain injury showed that it presents neuroprotective effects due to its anti-inflammatory, antiapoptotic, and antioxidant activities, which is demonstrated by the decrease in myeloperoxidase (MPO) and caspase-3 levels as well as the increase in SOD activity [35].

All these studies demonstrate that meldonium can be considered an effective therapeutic approach in preventing MD and impaired energy homeostasis [8,32].

Meldonium as a cardioprotective drug in athletes

Practicing physical exercise has a beneficial effect on health; however, in the case of performance athletes, there is cardiac remodeling with left ventricular dilatation and reduced ejection fraction, known as athletic heart syndrome. Differential diagnosis to distinguish between sports-induced cardiac modifications and pathological conditions is often difficult to achieve. If conditions like hypertrophic cardiomyopathy, dilated cardiomyopathy, or arrhythmogenic ventricular cardiomyopathy are not correctly diagnosed in athletes, it can lead to sudden cardiac arrest and death during training or sports competition. On the other hand, unnecessary restrictions on athlete training and competition can have disastrous consequences for the athlete [36].

Normally, a significant amount of ATP in cardiac tissue comes from fatty acid β-oxidation. However, during physical exertion, hypoxia in myocardial cells leads to incomplete mitochondrial fatty acid oxidation and the accumulation of acyl-carnitines, which can cause cellular membrane degradation. Studies have shown an accumulation of acyl-carnitines, especially palmitoyl-carnitine, in patients with cardiovascular diseases, and the acyl-carnitine/free carnitine ratio is a biomarker in heart failure [37,38].

Meldonium is used as an anti-anginal medication in countries outside the European Union because it prevents post-infarction ischemia and the progression of atherosclerosis. It improves cardiac functional parameters and reduces the incidence and severity of arrhythmias [39,40,41]. Dysregulation of fatty acid metabolism in right ventricular failure is caused by the reduction in the expression of PGC1α and PPARδ, which targets the genes responsible for producing critical enzymes in fatty acid β-oxidation. Meldonium activates the PPARδ/PGC1α pathway and decreases ROS production in mitochondria and the risk of MD, which in turn may contribute to a reduction in cardiac injury [34]. In a rat model of diabetes mellitus, meldonium facilitates post-ischemic cardiac recovery by promoting glucose metabolism instead of fatty acids by increasing the activity of pyruvate dehydrogenase and reducing acyl-carnitine concentration [42,43].

In the current literature, few studies link meldonium to sports performance and cardioprotective effects in humans. Dzerve et al. investigated the impact of meldonium on the peripheral circulation in patients with chronic heart failure. According to their study, meldonium combined with standard therapy was more effective than standard therapy alone in treating primary symptoms of chronic heart failure, improving exercise capacity, enhancing quality of life, and increasing peripheral circulation and vasodilation of both marginal and resistance vessels at rest and during exercise [44]. Another randomized, double-blind, placebo-controlled phase 2 trial assessed the efficacy of various doses of meldonium combined with standard therapy on the exercise tolerance of patients with stable angina pectoris. The study evaluated 512 patients, all of whom had coronary artery disease and positive exercise tests for ischemia. The results demonstrated that the improvement in exercise tolerance due to meldonium in patients with stable angina was dose-dependent [45]. Figure 3 aims to provide a clear visual representation of the cardioprotective mechanism of meldonium in hypoxic conditions induced by effort.

## Conclusions

Meldonium appears on the WADA list of prohibited substances during competition for professional athletes due to its metabolic modulating effect between the use of fatty acids and glucose for energy production. It is known that the maximal effort capacity when using carbohydrates is superior to that of lipids. However, the pharmacological mechanism implicated in providing unfair advantages in sports can bring real health benefits to athletes. By competitively inhibiting de novo carnitine synthesis and its absorption from dietary sources, meldonium reduces parameters of oxidative stress and inflammation, which contribute to MD. Additionally, by redirecting metabolism of fatty acids to a carnitine-independent one in the peroxisomes, it reduces the formation of acyl/acetyl-carnitines implicated in cardiac pathology potentially protecting against vascular trauma in ultra-athletes.
